# D-ribose-loaded hydrogel modulates necrosis progression and wound stability in a rabbit random-pattern skin flap model

**DOI:** 10.1016/j.jpra.2025.12.033

**Published:** 2026-01-03

**Authors:** Khalil Rostami, Omid Zehtab, Helia Ghorbani, Mahdi Mohebbi, Payam Asadi Ghahnaviyeh, Parisa Haghi, Sayed Esmaeil Tabatabaei

**Affiliations:** a Department of Plastic and Reconstructive Surgery, Shahid Beheshti University of Medical Sciences, Tehran, Iran; b Faculty of Veterinary Medicine, Tehran University of Medical Sciences, Tehran, Iran; c School of Medicine, Shahid Beheshti University of Medical Sciences, Tehran, Iran; d Department of General Surgery, Shahid Beheshti University of Medical Sciences, Tehran, Iran; e Department of Surgery, Zanjan University of Medical Sciences, Zanjan, Iran

**Keywords:** D-ribose, Hydrogel, Random-pattern skin flap, Ischemia, Necrosis progression, Wound dehiscence

## Abstract

**Background:**

Random-pattern skin flaps remain highly susceptible to ischemic injury, resulting in progressive distal necrosis and wound instability. Early metabolic failure is a central component of ischemia–reperfusion injury in flap tissue. D-ribose, a pentose sugar involved in adenine nucleotide and adenosine triphosphate (ATP) resynthesis, may support metabolic recovery in ischemic environments. This study investigated whether local delivery of a D-ribose-loaded hydrogel modulates necrosis progression and wound stability in a rabbit random-pattern skin flap model.

**Methods:**

Twenty-six adult male New Zealand White rabbits were randomly assigned to receive either a D-ribose-loaded chitosan hydrogel or an identical ribose-free control. A standardized caudally based random-pattern dorsal skin flap was elevated in each animal. Flap necrosis percentage, absolute necrotic area, and wound dehiscence were assessed using standardized digital imaging on postoperative days 3, 7, 9, 11, and 14. Flap failure was defined as development of ≥50% necrosis. Longitudinal changes were analyzed using linear mixed-effects modeling, while time-to-event outcomes were evaluated using Kaplan–Meier analysis with Cox regression. Dehiscence outcomes were analyzed using logistic regression and analysis of covariance.

**Results:**

D-ribose hydrogel significantly modulated the temporal progression of flap necrosis, with a distinct group × time interaction compared with controls (*p* = 0.038). Treatment delayed progression to flap failure, increasing the median time to ≥50% necrosis from 7 to 11 days (hazard ratio 0.38; 95% CI 0.17–0.85; *p* = 0.018). Wound dehiscence occurred less frequently in the D-ribose group than in controls (15.4% vs. 61.5%; odds ratio 0.12; 95% CI 0.02–0.78; *p* = 0.015), and dehiscence areas were significantly smaller. Mean necrosis percentage correlated positively with dehiscence extent (Spearman’s ρ = 0.68; *p* < 0.001).

**Conclusion:**

Local delivery of a D-ribose-loaded hydrogel modulated necrosis progression and improved wound stability in a rabbit random-pattern skin flap model. These findings support metabolically active hydrogel-based strategies as a promising adjunct to enhance flap reliability under ischemic conditions.

## Introduction

Random-pattern skin flaps remain essential reconstructive tools when primary closure or skin grafting is not feasible.^1^, ^2^, ^3^ Their vascular supply relies exclusively on the subdermal plexus, resulting in a proximal-to-distal perfusion gradient that places the distal portion at greatest risk of ischemic necrosis.^2^^,^^3^ Distal flap loss continues to compromise reconstructive outcomes by causing wound dehiscence, infection, delayed healing, and the need for secondary procedures.^3^

Ischemia–reperfusion (I/R) injury is a principal contributor to distal necrosis in random-pattern flaps.^4^, ^5^, ^6^ During ischemia, cellular ATP depletion and metabolic failure occur, while reperfusion triggers oxidative stress, endothelial dysfunction, and microvascular obstruction that preferentially impair distal flap perfusion.^4^, ^5^, ^6^ Despite extensive experimental investigation into surgical delay techniques and pharmacologic modulation, clinically practical and locally applicable strategies that reliably improve distal flap survival remain limited.^7^, ^8^, ^9^

Because early I/R injury is characterized by metabolic collapse, localized metabolic support represents an underexplored therapeutic approach. D-ribose is a metabolizable pentose sugar that facilitates adenine nucleotide and ATP resynthesis through the pentose phosphate pathway.^10^, ^11^, ^12^ Although existing evidence derives primarily from ischemic cardiac models, ATP depletion is a conserved feature of ischemic injury in metabolically active soft tissues, including skin.^4^^,^^13^ This shared vulnerability provides a biologically coherent rationale for evaluating D-ribose as a localized adjunct in ischemia-prone skin flaps.

Topical delivery offers the advantage of concentrating D-ribose at the site of ischemia while minimizing systemic exposure; however, the local biological effects of D-ribose in random-pattern flap models have not been systematically evaluated.^14^ This study investigated whether topical D-ribose delivered within a hydrogel matrix improves survival of a standardized caudally based dorsal random-pattern skin flap in a rabbit model.

## Methods

### Study design and ethical approval

This randomized, controlled, parallel-group experimental study was conducted using a rabbit dorsal skin flap model. The study protocol was approved by the Institutional Animal Care and Use Committee of Shahid Beheshti University of Medical Sciences (IR.SBMU.AEC.1404.002). All procedures were performed in accordance with the ARRIVE 2.0 guidelines and internationally accepted standards for the care and use of laboratory animals.^13^

### Animals and housing

Twenty-six healthy adult male New Zealand White rabbits (aged 4–6 months, weighing 2.5–3.5 kg) were included. Animals were acclimatized for 1 week before surgery under controlled laboratory conditions (temperature 21–23°C, humidity 45–55%, 12-h light/dark cycle), with free access to standard chow and water. Only clinically healthy animals, as confirmed by veterinary examination, were enrolled, in accordance with established experimental wound-healing models.^15^

### Randomization and blinding

Animals were randomly allocated into two equal groups (*n* = 13 per group) using a computer-generated randomization sequence. The intervention group received D-ribose–loaded chitosan hydrogel, whereas the control group received an identical chitosan hydrogel without D-ribose. Owing to the nature of the intervention, the operating surgeon was not blinded to group allocation. Outcome assessment and quantitative image analyses were performed by an investigator blinded to group assignment.

### Preparation of D-ribose chitosan hydrogel

Hydrogels were prepared using an ionic gelation method. Briefly, chitosan (3 g; degree of deacetylation > 85%; medium molecular weight 100–300 kDa; Fine-nano, Iran) was dissolved in 100 mL of 1% (v/v) acetic acid under magnetic stirring (700 rpm) for 2 h at room temperature until a clear solution was obtained. The solution was centrifuged (5000 × *g*, 10 min) to remove air bubbles and sterilized by filtration through a 0.22 µm membrane filter.

D-ribose powder (2.5 g; ≥ 98% purity; Nobel Chemical, Iran) was added to the chitosan solution and stirred for an additional 30 min. Crosslinking was achieved by gradual dropwise addition of sodium tripolyphosphate (TPP; 2% w/v) under gentle stirring for 30 min, as commonly used for chitosan-based hydrogels.^16^

The resulting hydrogel was rinsed with deionized water, and the final pH was adjusted to 6.5–7.0 using phosphate-buffered saline. The control hydrogel was prepared using the same protocol without D-ribose. All hydrogels were stored in sterile containers at 4 °C until use. The use of sugar-based hydrogels for local tissue support was informed by prior experimental wound-healing studies.^17^

### Surgical procedure and intervention

Following induction of general anesthesia with intramuscular ketamine (40 mg/kg) and xylazine (4 mg/kg), the dorsal region was shaved and prepared under sterile conditions. A caudally based random-pattern dorsal skin flap measuring 10 × 3 cm was designed and marked on the mid-dorsal region, based on established random-pattern flap models.^18^^,^^19^

Skin incisions were made along the cranial and lateral borders, and the flap was elevated using sharp dissection in a plane deep to the panniculus carnosus, leaving the flap attached exclusively at its caudal base. Particular attention was paid to preserving the integrity of the caudal pedicle throughout flap elevation. Meticulous hemostasis was achieved using bipolar cautery.

After complete elevation, the flap was temporarily reflected to allow intervention at the flap bed. In the intervention group, approximately 1 mL of sterile D-ribose–loaded chitosan hydrogel was applied to the subcutaneous surface of the flap bed to achieve broad and uniform coverage while avoiding accumulation beneath the caudal pedicle, in order to prevent local compression and impaired microcirculation, as previously recommended.^17^ Animals in the control group underwent an identical procedure using hydrogel without D-ribose.

**Figure 1:**
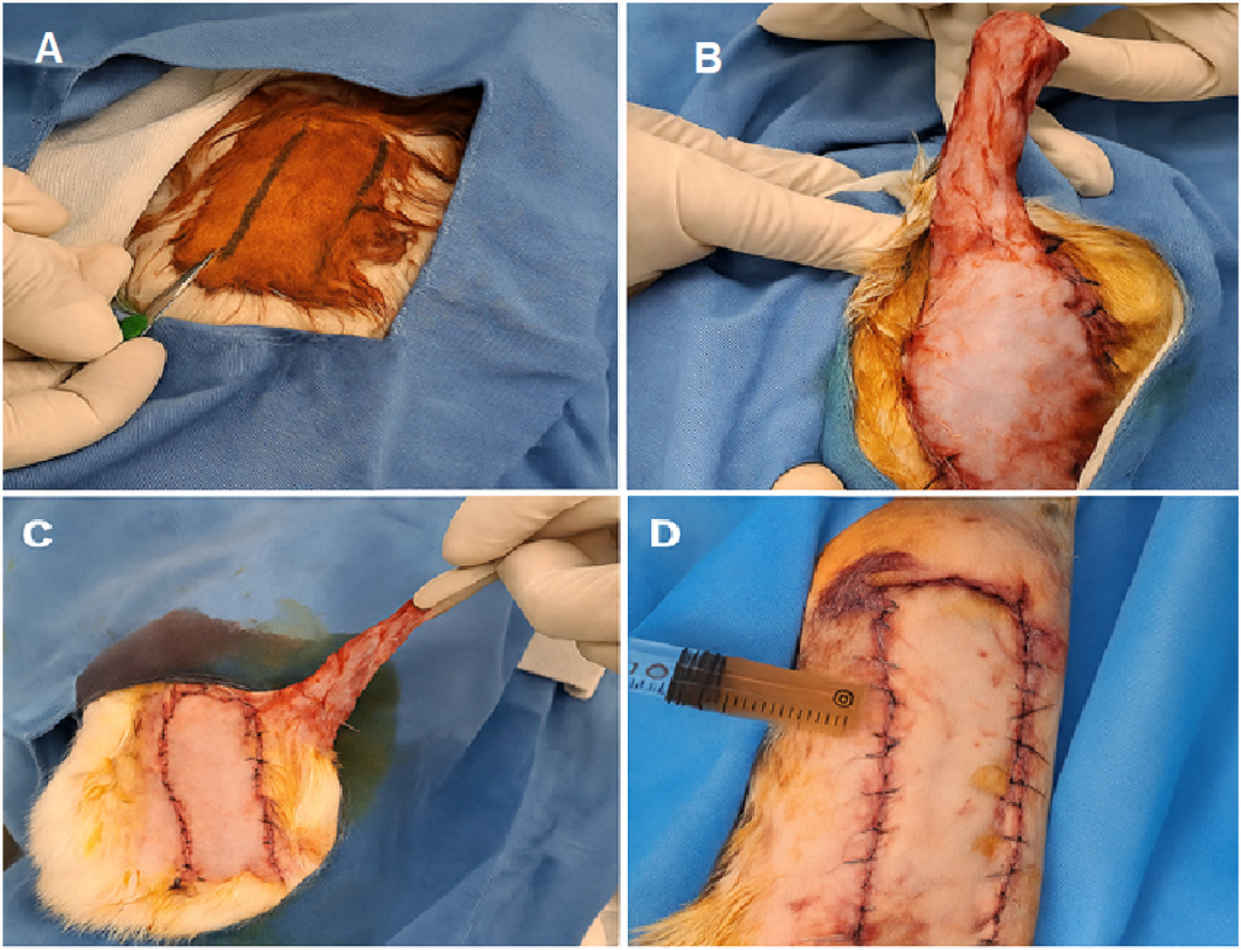
Stepwise surgical procedure of a caudally based random-pattern dorsal skin flap in the rabbit model. (A) Preoperative marking of a 10 × 3 cm caudally based dorsal skin flap on the mid-dorsal region following shaving and sterile skin preparation. (B) Elevation of the dorsal skin flap in a plane deep to the panniculus carnosus, with careful preservation of the caudal pedicle. (C) Temporary reflection of the elevated flap, exposing the underlying flap bed prior to intervention. (D) Flap repositioning and closure using interrupted simple sutures with 4-0 nylon, followed by focal subcutaneous injection to ensure uniform distribution of the applied hydrogel and to prevent leakage or pooling beneath the caudal base.

**Figure 2:**
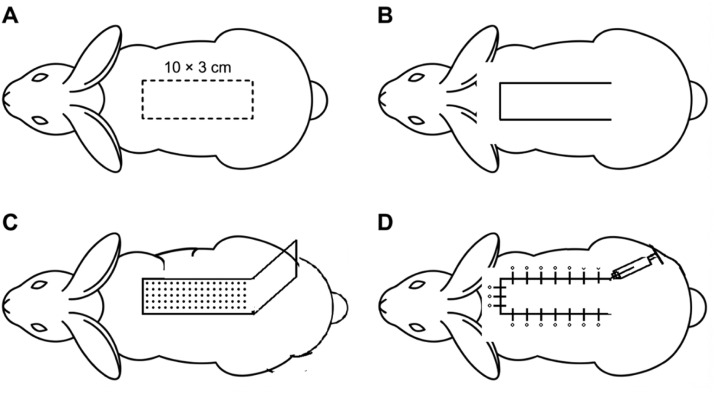
Schematic illustration of the rabbit caudally based random-pattern dorsal skin flap model and intervention approach. (A) Design and dimensions (10 × 3 cm) of the caudally based dorsal skin flap on the mid-dorsal region. (B) Longitudinal incision lines defining the flap borders. (C) Elevation of the random-pattern flap with preservation of the caudal pedicle. (D) Medical illustration of hydrogel application and subcutaneous delivery within the flap bed prior to flap repositioning and closure.

The flap was then repositioned to its original location and secured using interrupted simple sutures with 4-0 nylon, with slight wound-edge eversion to minimize tension and buttonhole ischemia. To ensure homogeneous hydrogel distribution and to prevent leakage or pooling, additional focal subcutaneous injections were performed after flap closure. A non-adherent dressing was applied at the end of the procedure. The sequential steps of flap elevation, intervention, and closure are documented with representative intraoperative photographs in Figure 1 and illustrated schematically in Figure 2.

### Postoperative care and euthanasia

Postoperative analgesia consisted of meloxicam (1–1.5 mg/kg, subcutaneous, once daily) and buprenorphine (0.05 mg/kg, subcutaneous, as needed) for 48–72 h, following established multimodal analgesia protocols in rabbits.^20^ Animals were monitored daily for general condition, wound appearance, signs of infection, dehiscence, or flap necrosis. Humane endpoints were predefined, and animals showing uncontrollable pain, severe complications, or extensive necrosis were withdrawn from the study.

At the end of the observation period (postoperative day 14), animals were humanely euthanized in accordance with the AVMA Guidelines for the Euthanasia of Animals (2020) using an overdose of anesthetic agents administered under veterinary supervision.^21^

### Outcome measures and image analysis

The primary outcome was flap necrosis percentage. Secondary outcomes included absolute necrotic area, flap survival percentage, and wound dehiscence incidence and area.

Standardized digital photographs were obtained on postoperative days 3, 7, 9, 11, and 14, with a reference scale included in each image. Images were analyzed using ImageJ software (National Institutes of Health, USA), a validated tool for quantitative planimetric analysis.^22^ Total flap area and necrotic area were manually delineated, and necrosis percentage was calculated as:

$$\text{Necrosis}\left(\%\right)=\frac{\text{Necroticarea}}{\text{Total}\;\text{flap}\;\text{area}}\times 100$$

### Statistical analysis

Analyses were performed using a two-sided significance level of 0.05. Continuous variables are reported as mean ± SD or median (IQR), as appropriate. Normality was assessed using the Shapiro–Wilk test and Q–Q plots.

Longitudinal changes in necrosis percentage were analyzed using a linear mixed-effects model with fixed effects for group, time, and group × time interaction, and a random intercept for each animal to account for within-subject correlation and missing repeated measurements. Between-group comparisons at postoperative day 14 were performed using independent-samples t-tests or Mann–Whitney U tests, depending on data distribution. Dehiscence incidence was compared using Fisher’s exact test.

## Results

### Flap necrosis

**Figure 3:**
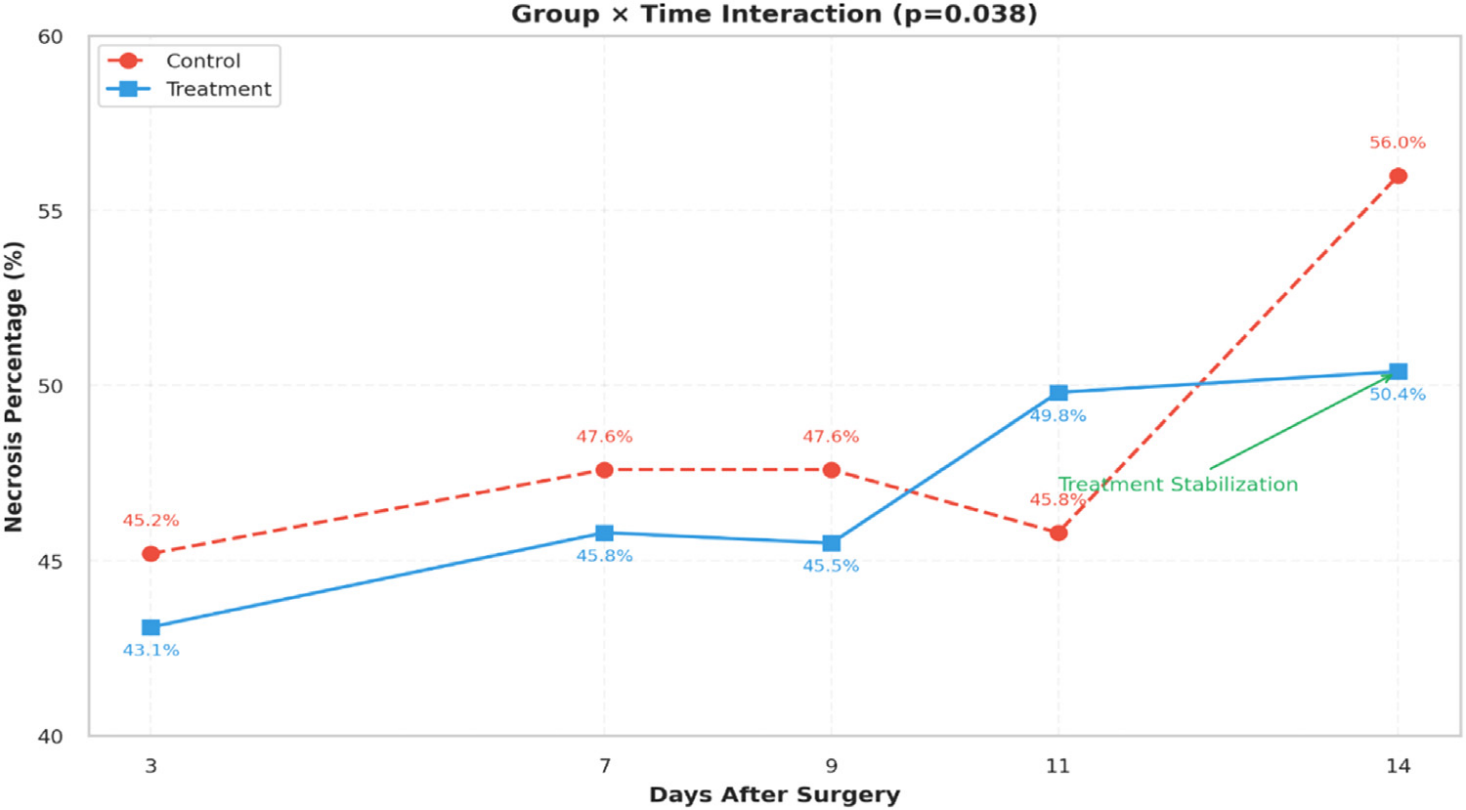
Temporal progression of flap necrosis. Mean necrosis percentage (± SEM) in the D-ribose and control groups measured on postoperative days 3, 7, 9, 11, and 14. A linear mixed-effects model demonstrated a significant group × time interaction, indicating divergent trajectories of necrosis progression between groups.

The longitudinal progression of flap necrosis differed significantly between groups, with a more stable temporal pattern observed in the D-ribose group compared with controls (Figure 3). Linear mixed-effects modeling demonstrated a significant group × time interaction (*p* = 0.038), indicating divergent trajectories of necrosis progression over the postoperative period (Table 1).

**Table 1:** Primary necrosis-related outcomes comparing the D-ribose and control groups.

Outcome measure	D-ribose group	Control group	Statistical estimate	*p*-value
Final necrosis percentage (POD 14)	50.35 ± 10.53%	56.03 ± 9.43%	Cohen’s *d* = −0.49	0.049
Group × time interaction (LMM)	—	—	Estimate = −0.217 (SE 0.103), *t* = −2.107	0.038
Median time to ≥50% necrosis	11 days (95% CI 9–14)	7 days (95% CI not estimable)	HR = 0.38 (95% CI 0.17–0.85)	0.018

Primary necrosis-related outcomes in the D-ribose and control groups. Continuous variables are presented as mean ± SD unless otherwise specified. Longitudinal changes were analyzed using a linear mixed-effects model with fixed effects for group, time, and group × time interaction. Time-to-event outcomes were analyzed using Kaplan–Meier estimates and Cox proportional hazards modeling.

Time-to-event analysis showed that D-ribose treatment delayed progression to flap failure, defined as development of ≥50% necrosis. The median time to flap failure was 11 days (95% CI 9–14) in the D-ribose group and 7 days in the control group. This corresponded to a 62% reduction in hazard compared with controls (hazard ratio 0.38; 95% CI 0.17–0.85; Table 1).

### Wound dehiscence

The incidence of wound dehiscence was significantly lower in the D-ribose group than in the control group (1/13 [15.4%] vs. 5/13 [61.5%]). Logistic regression analysis demonstrated a significantly reduced odds of dehiscence in the D-ribose group (odds ratio 0.12; 95% CI: 0.02–0.78; *p* = 0.015; Table 2).

**Table 2:** Comparison of wound dehiscence outcomes between study groups.

Outcome measure	D-ribose group	Control group	Statistical estimate	*p*-value
Incidence of dehiscence	1/13 (15.4%)	5/13 (61.5%)	OR = 0.12 (95% CI 0.02–0.78)	0.015
Dehiscence area (mm²)	47.3 ± 22.1	94.6 ± 38.2	Mean difference = −47.3 (95% CI −72.1 to −22.5)	<0.001

Comparison of wound dehiscence outcomes between the D-ribose and control groups. Dehiscence incidence was analyzed using logistic regression. Dehiscence area was compared using analysis of covariance (ANCOVA) adjusting for initial flap area.

Among animals in which dehiscence occurred, the extent of wound separation was significantly smaller in the D-ribose group. Analysis of covariance adjusting for initial flap area confirmed a reduced mean dehiscence area compared with controls (adjusted mean difference = −47.3 mm²; 95% CI: −72.1 to −22.5; *p* < 0.001; Table 2).

### Time-to-event analysis: flap failure (necrosis ≥50%)

**Figure 4:**
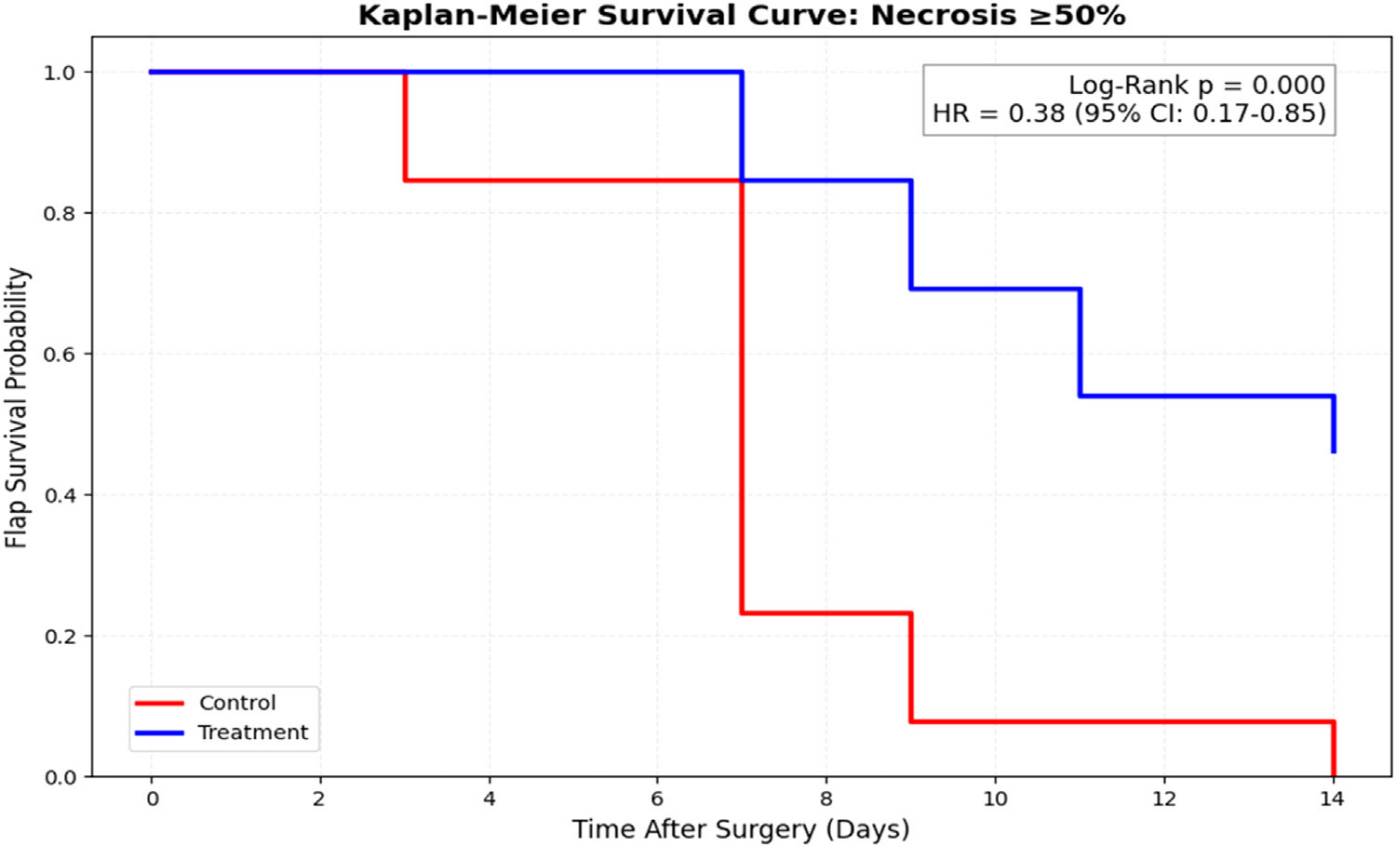
Kaplan–Meier analysis of flap survival. Kaplan–Meier curves illustrating time to flap failure, defined as development of ≥50% necrosis. D-ribose treatment significantly delayed flap failure compared with controls (log-rank *p* = 0.011).

Kaplan–Meier survival analysis demonstrated a significant difference in time to flap failure between groups (Figure 4). Animals treated with D-ribose exhibited prolonged flap survival compared with controls (log-rank *p* = 0.011).

Cox proportional hazards modeling confirmed a reduced hazard of flap failure in the D-ribose group (hazard ratio = 0.38; 95% CI: 0.17–0.85; *p* = 0.018).

### Association between necrosis and dehiscence

A significant positive association was observed between mean necrosis percentage and maximum dehiscence area across animals. Spearman’s rank correlation coefficient was ρ = 0.68 (95% CI: 0.42–0.84; *p* < 0.001), indicating that greater tissue necrosis was associated with more extensive wound dehiscence.

## Discussion

This randomized controlled experimental study shows that local delivery of D-ribose via a chitosan hydrogel modifies the temporal course of ischemic injury in random-pattern skin flaps. Rather than producing a large absolute reduction in final necrotic burden, D-ribose treatment primarily altered necrosis kinetics, delayed progression to flap failure, and improved wound stability, as reflected by a lower incidence of wound dehiscence.

This distinction is clinically relevant. In random-pattern flaps, distal ischemia is often unavoidable because of intrinsic limitations in subdermal plexus perfusion.^1^, ^2^, ^3^ Consequently, interventions that stabilize ischemic tissue and delay irreversible injury, even without fully eliminating necrosis, may still translate into meaningful improvements in reconstructive outcomes.

The significant group × time interaction observed in longitudinal analysis indicates that D-ribose altered the trajectory of tissue injury rather than affecting only the final endpoint. This aligns with current concepts of flap failure as a dynamic, time-dependent process driven by ischemia–reperfusion injury, microvascular dysfunction, and metabolic exhaustion.^4^, ^5^, ^6^

Hydrogel-based strategies increasingly aim to modulate the ischemic microenvironment rather than provide passive coverage or single-target rescue. In this context, biomaterials designed to buffer oxidative stress and support local metabolic demands have been proposed to prolong tissue viability during critical ischemic windows, even when distal necrosis ultimately occurs.^23^ Flap-specific evidence also supports this paradigm: Ju et al. reported improved random-pattern flap outcomes with hydrogel-mediated microenvironmental modulation linked to endothelial preservation and limitation of secondary ischemic expansion.^24^ The present findings extend this framework by suggesting that supplementation with a simple metabolic substrate, delivered locally within a hydrogel matrix, may enhance early ischemic tolerance during the postoperative period.

The biological plausibility of D-ribose as a metabolic support agent lies in its role as a precursor for phosphoribosyl pyrophosphate and adenine nucleotide resynthesis. ATP depletion is a conserved hallmark of ischemic injury across tissues, including skin.^4^^,^^25^ Although much of the D-ribose literature derives from cardiovascular settings, these studies consistently report accelerated restoration of high-energy phosphate pools following ischemic stress.^10^, ^11^, ^12^ In the present study, the data support a localized metabolic support hypothesis in which D-ribose, delivered to the flap bed (with adjunct focal delivery after closure), enhances tissue tolerance to ischemic stress during the early phase of injury rather than implying direct extrapolation from systemic cardiac effects.

One of the most consistent findings was the pronounced reduction in wound dehiscence. Dehiscence is not merely a mechanical complication; it reflects underlying tissue viability, collagen integrity, and microvascular sufficiency.^17^ The strong correlation between necrosis extent and dehiscence observed here reinforces the concept that tissue survival and wound mechanics are tightly coupled. By delaying necrosis progression, D-ribose treatment may have preserved functional wound-edge integrity during the critical early healing phase.

The modest difference in final necrosis percentage observed in this study is consistent with previous investigations of random-pattern skin flaps, in which distal tissue is intrinsically predisposed to ischemic loss owing to limitations of subdermal plexus perfusion.^8^^,^^19^ Accordingly, the principal therapeutic value of D-ribose hydrogel appears to lie in stabilizing early healing dynamics and improving wound integrity, rather than in completely preventing distal necrosis.

This study has limitations. Tissue ATP levels, angiogenic markers, and oxidative stress parameters were not directly measured, limiting mechanistic inference regarding metabolic and microvascular pathways. In addition, hydrogel release kinetics and degradation behavior were not quantified. Nevertheless, the study was designed around predefined outcomes and used longitudinal modeling to capture time-dependent injury patterns.

Future studies should incorporate molecular and histological assessments to validate metabolic and microvascular mechanisms, alongside release-kinetic characterization of the formulation. Dose–response experiments and evaluation in larger animal models will be important prior to clinical translation. Overall, the present findings provide a rationale for further investigation of metabolically active, locally delivered hydrogels as adjuncts in flap surgery.

## Conclusions

Local delivery of D-ribose–loaded chitosan hydrogel altered the temporal course of ischemic injury in random-pattern skin flaps by delaying necrosis progression and improving wound stability. These findings support metabolic support as a viable adjunctive strategy in flap surgery and position D-ribose hydrogel as a promising, translationally relevant therapeutic platform.

## Ethical approval

Approved by the Institutional Animal Care and Use Committee of Shahid Beheshti University of Medical Sciences (IR.SBMU.AEC.1404.002).

## Funding

This research did not receive any specific grant from funding agencies in the public, commercial, or not-for-profit sectors.

## Data availability

The data that support the findings of this study are available from the corresponding author upon reasonable request.

## Declaration of competing interest

The authors declare that they have no known competing financial interests or personal relationships that could have appeared to influence the work reported in this paper.

## References

[1] Wong V.W., Gurtner G.C., Longaker M.T., Townsend C.M., Beauchamp R.D., Evers B.M., Mattox K.L. (2022). Sabiston Textbook of Surgery.

[2] Morris S.F., Taylor G.I. (1995). The direct and indirect vascular supply to the skin and underlying tissues. Plast Reconstr Surg.

[3] Rehim S.A., Chung K.C., Janis J.E. (2014). Essentials of Plastic Surgery.

[4] Eltzschig H.K., Eckle T. (2011). Ischemia and reperfusion—From mechanism to translation. Nat Med.

[5] Kalogeris T., Baines C.P., Krenz M., Korthuis R.J. (2012). Cell biology of ischemia–reperfusion injury. Int Rev Cell Mol Biol.

[6] Kalogeris T., Korthuis R.J. (2012). Reperfusion injury and reactive oxygen species in cutaneous ischemia. Microcirculation.

[7] Lee J.T., Chen D.S., Chen K.S., Chen T.M. (2019). Strategies to enhance skin-flap viability: current evidence and future directions. J Plast Surg Hand Surg.

[8] Dunn R.M., Mancoll J.S. (1992). Flap physiology: direct and indirect delay mechanisms. Plast Reconstr Surg.

[9] Kinoshita M., Tsunoda S., Kimura S., Nakagawa I., Matsumoto K. (2014). Nitric oxide modulation and flap survival: biochemical and physiological evidence. Ann Plast Surg.

[10] Zimmer H.G. (1989). Regulation of myocardial energy metabolism: the role of D-ribose. Curr Ther Res.

[11] Yamada K.A., Katz A. (1988). D-ribose and adenine nucleotide metabolism in the heart. Basic Res Cardiol.

[12] Pliml W., von Arnim T., Stablein A., Hofmann H., Zimmer H.G. (1992). Effects of D-ribose on exercise-induced ischemia in stable coronary artery disease. Lancet.

[13] Percie du Sert N. (2020). The ARRIVE guidelines 2.0: updated guidelines for reporting animal research. PLoS Biol.

[14] Singh V.P., Bali A., Singh N., Jaggi A.S. (2014). Advanced glycation end products and diabetic complications. Korean J Physiol Pharmacol.

[15] Grada A., Mervis J., Falanga V. (2018). Research techniques made simple: animal models of wound healing. J Invest Dermatol.

[16] Calvo P., Remuñán-López C., Vila-Jato J.L. (1997). Novel hydrophilic chitosan–polyethylene oxide nanoparticles as protein carriers. J Appl Polym Sci.

[17] Dikici S., Yar M., Bullock A.J., Shepherd J., Roman S., MacNeil S. (2021). Developing wound dressings using 2-deoxy-D-ribose to induce angiogenesis as a backdoor route for stimulating the production of vascular endothelial growth factor. Int J Mol Sci.

[18] McFarlane R.M., DeYoung G., Henry R.A. (1965). The design of a pedicle flap in the rat to study necrosis and its prevention. Plast Reconstr Surg.

[19] Lee J.H., You H.J., Lee T.Y., Kang H.J. (2022). Current Status of Experimental Animal Skin Flap Models: Ischemic Preconditioning and Molecular Factors. Int J Mol Sci.

[20] Goldschlager G.B., Gillespie V.L., Palme R., Baxter M.G. (2013). Effects of multimodal analgesia with buprenorphine and meloxicam in rabbits. J Am Assoc Lab Anim Sci.

[21] American Veterinary Medical Association (AVMA). AVMA Guidelines for the Euthanasia of Animals: 2020 Edition. 2020. Available at https://www.avma.org/sites/default/files/2020-02/Guidelines-on-Euthanasia-2020.pdf

[22] Schneider C.A., Rasband W.S., Eliceiri K.W. (2012). NIH image to ImageJ: 25 years of image analysis. Nat Methods.

[23] Wang W., Tai S., Tao J. (2024). Innovative hydrogel-based therapies for ischemia-reperfusion injury: bridging the gap between pathophysiology and treatment. Mater Today Bio.

[24] Ju Y., Yang P., Liu X. (2024). Microenvironment remodeling self-healing hydrogel for promoting flap survival. Biomater Res.

[25] Prasad S.S., Wu G., Chen J. (2009). Mitochondrial dysfunction and metabolic insufficiency in cutaneous ischemia. Plast Reconstr Surg.

